# A Perspective on Intimate Partner Violence Since COVID-19

**DOI:** 10.3389/fgwh.2021.788061

**Published:** 2021-12-08

**Authors:** Raveed Khan, Syriah David

**Affiliations:** Department of Paraclinical Sciences, University of the West Indies, St. Augustine, Trinidad and Tobago

**Keywords:** partner abuse, COVID-19 pandemic, intimate abuse, Trinidad and Tobago, primary care (MeSH)

The advent of the COVID-19 pandemic saw the introduction of many unprecedent actions occurring within our communities and by extension our country. Whilst many of us may have heard the term pandemic before, few would have had the knowledge or experience of the measures that were taken to contain coronavirus in our country. The introduction of lockdown measures such as the closure of public places like bars restaurants, malls, places of worship, gyms, spas and beauty services, casinos, cinemas (1); as well as, stay-at-home orders like working from home for non-essential persons and closure of all schools were just a few of the measures that were put into place to help curb the spread of COVID-19. The ramifications of these measures would pan out over time with some becoming apparent earlier than others and with grave consequences, particularly, an increase in acts of intimate partner violence.

Intimate partner violence is sometimes referred to as domestic violence since a large number of acts committed by perpetrators occur in the home. Locally, in Trinidad and Tobago prevalence rates as high as 41% have been reported with alcohol and drug abuse being cited as the leading cause (2). In 2017 alone the Trinidad and Tobago Police Service (TTPS) reported that there were 1,100 reports of domestic violence with 43 murders linked to domestic violence. According to the August 2020 report from the Trinidad and Tobago Central Registry on Domestic Violence, there were 7,594 reports related to domestic violence between the period 2014 to 2019. Approximately 75% of these reports were related to female victims. In 2019, there were 232 reports of domestic violence with 81% of reports being made by women, where 48.5% were between 30 to 49 years of age and 22.5% of women were between 18 to 29 years of age. In 2020, there were 556 cases of domestic violence that were reported. During the period of January to March 2021, there were 826 reports of domestic violence (3).

Based on these findings it would appear that COVID-19 created a milieu conducive for a domestic violence surge with the background of an existing high prevalence rate compounded by confinement and possibly increased consumption of alcohol and drugs at home. The Centres for Disease Control and Prevention (CDC) highlights individual, relational, community and societal risk factors for domestic violence. These risk factors include common themes such as heavy alcohol and drug use, economic stress (for example, unemployment), having few friends, being isolated from other people and a desire for power and control in relationships (4). Alcohol use/abuse in particular has been associated with male-to-female partner violence (5). Alcohol plays a key role in the power and control wheel concept by increasing the user's sense of personal power and domination over others. An increased sense of power and control can, in turn, make it more likely that an abuser will attempt to exercise that power and control over another (6).

The power and control wheel can also be turned through isolation and economic abuse. Isolation of the victim is further enhanced by stay-at-home measures aimed at preventing the spread of COVID-19, but also trapping victims of domestic violence with their abusers. Notwithstanding communication technologies, there are also physical constraints on the victim preventing them from accessing direct in person support and retreat to the safety of family, friends and shelters. Behaviours such as constant surveillance and enforcement of rigid rules by perpetrators serve to fuel the isolation and propagate acts of violence in the home. The potential for economic abuse has also increased since the pandemic due to the effects of job-losses, furloughs, reduced working hours and general declines in economic activity. Consequently perpetrators' financial stresses coupled with the propensity to consume alcohol as an escape, create a highly charged home environment with acts of violence becoming likely possibilities. In addition, some victims of domestic violence are financially dependent on their abusers, therefore making it difficult for them to leave these home environments. Local data reported by UN Women suggests that as of December 2020, only 39.3% of indicators needed to monitor the sustainable developmental goals (SDGs) from a gender perspective were available. There were gaps in key areas such as unpaid care and domestic work, and information on the gender pay gap. However, it can be noted that the unemployment rate among women over 15 years of age was found to be 6.3% compared to 4.6% of men in the same age range (7).

The concept of male privilege may have also been strengthened during the COVID-19 pandemic as men occupying supervisory and managerial roles at their workplaces have become day to day bosses at home thereby increasing their tendency to act like masters of the castle and relegating the woman's role to that of a servant. Indeed, this domination and control through the use of male privilege plays a significant role in male abusers who often consider abusive behaviour as a right and a privilege. A Trinidadian qualitative report revealed that male dominance in the family is a key factor creating a risk of violence. Controlling behaviours such as isolation tactics, accusations, threats and stalking also highlight the relationship between male dominance in the family and multiple, intersecting forms of violence. Accusation by partners is common, repetitive and used to precipitate threatening, hitting, slapping, cursing and choking (8).

Tactics used to maintain power and control through the use of male privilege in the context of COVID-19 were highlighted by the Battered Women's Justice Project. These tactics include violation of others' personal space, using all the work and study areas in the home, disrupting routines and refusing to share the computer (10).

The COVID-19 pandemic in itself can be leveraged by perpetrators. Indeed those suffering from domestic violence may be less inclined to go to the hospital on account of fear of infection. Ultimately, the social distancing, albeit essential to contain COVID-19, may exacerbate the violence and maintain it less visible (9). Usher et al. also reported that COVID-19 is used as a coercive control mechanism whereby perpetrators exert further control in an abusive relationship, specifically in the use of containment, fear, and threat of contagion as a mechanism of abuse (11).

Increased acts of sexual violence have also been documented during this pandemic. A UK and Kenyan based study reported that Sexual and gender-based violence (SGBV), and particularly intimate partner violence (IPV), had spiked dramatically during the COVID-19 pandemic (12). India noted a surge of porn usage and sale of condoms and sex toys, reflecting increase in sexual activity thereby indirectly indicating increase in chances of sexual rights violation (13).

As a family physician, it is my view that we make the best use of the limited resources available to us to protect our population from the scourge of domestic violence that is accompanying the COVID-19 pandemic. Now more than ever comes the time for innovation and tact in the implementation of measures to screen, guide and act definitively to protect the victims of domestic violence. Toward this end, the WHO's ALIVES framework for inquiring and responding to a disclosure of domestic violence serves as a primer for facilitating disclosure, validating concerns and enhancing safety and support (14).

This framework can perhaps best be implemented through the use of telehealth which has now become a standard of care in order to mitigate against COVID-19. It is well-documented that women and children are the main seekers of health care. As health care providers, we must capitalise on that by tactfully screening for domestic violence during virtual encounters through the use of safe words or even advice on the wearing of certain colours of clothing as a signal that a survivor is concerned about their risk (15).

As a society we must never condone acts of domestic violence. Rather, we should seek to educate the victims about avenues for relief and try our best to dissuade perpetrators by creating a supportive environment especially in the era of COVID-19.

## Author Contributions

All authors listed have made a substantial, direct, and intellectual contribution to the work and approved it for publication.

## Conflict of Interest

The authors declare that the research was conducted in the absence of any commercial or financial relationships that could be construed as a potential conflict of interest.

## Publisher's Note

All claims expressed in this article are solely those of the authors and do not necessarily represent those of their affiliated organizations, or those of the publisher, the editors and the reviewers. Any product that may be evaluated in this article, or claim that may be made by its manufacturer, is not guaranteed or endorsed by the publisher.
